# Large-Scale SARS-CoV-2 Antigen Testing With Real-World Specimens

**DOI:** 10.3389/fpubh.2022.836328

**Published:** 2022-04-05

**Authors:** Ashish Parikh, Lauren Cooper, Daniel Frogel, Kerry Le Benger, Charles K. Cooper, Valentin Parvu

**Affiliations:** ^1^CityMD/Summit Medical Group, New York, NY, United States; ^2^Becton, Dickinson and Company, BD Life Sciences—Integrated Diagnostic Solutions, Sparks, MD, United States; ^3^George Mason University, School of Systems Biology, Manassas, VA, United States

**Keywords:** BD Veritor, COVID-19, SARS-CoV-2, rapid antigen testing, RT-PCR, real-world evidence (RWE), point-of-care

## Abstract

Real-world data are needed to establish SARS-CoV-2 rapid antigen testing (RAT) as an effective and reliable approach for SARS-CoV-2 screening. This study included 1,952,931 individuals who provided upper respiratory specimens during SARS-CoV-2 screening at CityMD urgent care locations in the New York metropolitan area from October 2020 to March 2021. Positive and negative results, as determined by the BD Veritor™ System for Rapid Detection of SARS-CoV-2 antigen (Veritor), were obtained for all individuals, with reflex reverse transcriptase-polymerase chain reaction (RT-PCR) testing performed on a case-by-case basis, per standard of care. Using verification bias adjustment, two alternative model assumptions were utilized for RAT results with missing reflex RT-PCR results. The worst antigen diagnostic performance estimates asserted that missing RT-PCR results would show a distribution similar to those RT-PCR results actually obtained, based on symptom category. The best antigen diagnostic performance estimates asserted that individuals without RT-PCR results had a clinical presentation consistent with RAT results, and, therefore, missing RT-PCR results would agree with RAT results. For patients with symptoms or high-risk exposure, 25.3% (*n* = 86,811/343,253) of RAT results were positive; vs. 3.4% (*n* = 53,046/1,559,733) positive for asymptomatic individuals without high-risk exposure. Reflex RT-PCR results were obtained from 46.3% (*n* = 158,836/343,253) and 13.8% (*n* = 215,708/1,559,733) of symptomatic and asymptomatic individuals, respectively. RT-PCR confirmed 94.4% (4,265/4,518) of positive and 90.6% (139,759/154,318) of negative RAT results in symptomatic individuals; and confirmed 83.4% (6,693/8,024) of positive and 95.3% (197,955/207,684) of negative RAT results in asymptomatic individuals. Applied assumptions for missing reflex RT-PCR results led to worst performance sensitivity estimates of 77.2 and 38.5% in the symptomatic and asymptomatic populations, respectively; assumptions for best performance estimates led to sensitivity values of 85.6 and 84.2%, respectively. Specificity values, regardless of assumptions or symptom category, ranged from 97.9–99.9%. At 10% SARS-CoV-2 prevalence, RAT positive predictive value was 86.9 and 99.0% for worst and best performance estimates across the total population, respectively; negative predictive values were >95% regardless of the applied assumption. Veritor test performance was consistent with that listed in the manufacturer instructions for use for symptomatic individuals. Real-world evidence should be gathered on RATs to support their efficacy as SARS-CoV-2 persists.

## Introduction

Coronavirus disease 2019 (COVID-19) can lead to severe respiratory illness and result in morbidity and mortality—especially for those with certain preexisting conditions (1). SARS-CoV-2, the virus that causes COVID-19, emerged at the end of 2019 (2) and has resulted in over 260 million COVID-19 cases and ~5 million COVID-19-related deaths worldwide. In the US, ~50 million COVID-19 cases and over 780,000 COVID-19 deaths have been recorded through November 2021. Within 8 months of the U.S. Food and Drug Administration's authorization of the first COVID-19 vaccine in December 2020, the weekly rates for both COVID-19 cases and case-relative mortalities in the US had dropped by over 90% (3). However, between June and July of 2021, the incident rate ratio of not-fully-vaccinated individuals to fully vaccinated individuals decreased at an unexpectedly high rate, suggesting that SARS-CoV-2 variants (e.g., Delta variant) were driving new cases (4). Worldwide SARS-CoV-2 variants pose a threat due to their increased ease of transmission and potential for increased disease severity—even in vaccinated or convalescent individuals (5).

Although vaccination is the most important tool to prevent the spread of SARS-CoV-2 (6), breakthrough cases in vaccinated individuals, along with transmission facilitated by unvaccinated individuals, necessitate continued SARS-CoV-2 testing in multiple settings (7). Nucleic acid amplification testing (NAAT) offers the highest commercial, analytical sensitivity for SARS-CoV-2 detection (8); however, rapid SARS-CoV-2 testing offers certain advantages over NAATs. Rapid antigen tests (RATs) provide a faster result turnaround time than reverse transcriptase-polymerase chain reaction (RT-PCR) (9). In addition, dedicated infrastructure and technical expertise are not required for RATs during point-of-care testing (10). Further, RATs have a better predictive value for infectiousness compared to RT-PCR (11), and recent epidemiological modeling shows that RAT-based surveillance testing, in combination with sequestration, can effectively reduce both disease burden and economic cost associated with the management of COVID-19 (12).

Recent work shows that performance values (e.g., test sensitivity and specificity) for individual SARS-CoV-2 RATs, reported from real-world studies, can differ when compared to those presented in the manufacturer's instructions for use (IFU) (13–18). Several factors, including study design and experimental bias, can affect RAT sensitivity estimates; (18) therefore, it is important to monitor their real-world performance (19). In addition, it is important to supplement registration studies with real-world studies to establish a precise performance profile for RATs over time. Here, we present real-world data from a large urgent care clinic network that includes over 1.95 million individuals undergoing testing for SARS-CoV-2 using the BD Veritor^TM^ System for Rapid Detection of SARS-CoV-2 (Veritor; Becton, Dickinson and Company; BD Life Sciences—Integrated Diagnostic Solutions, Sparks, MD, USA). Reflex RT-PCR testing was performed on a subset of individuals with Veritor results and analysis was conducted to determine the performance characteristics of this RAT (indication under FDA Emergency Use Authorization) across the study population.

## Methods and Materials

### Specimens

This retrospective study analyzed 1,952,931 million specimens from individuals screened for SARS-CoV-2 at CityMD Urgent Care Walk-in Medical Clinics in the New York metropolitan area (Queens, Brooklyn, Long Island, Bronx, Manhattan, Staten Island, Metro North, and New Jersey) between October 2020 and March 2021. Nasal swabs were utilized for Veritor testing and either nasal or nasopharyngeal swabs were collected for RT-PCR testing, according to clinic-specific standard of care (SOC). Symptoms associated with COVID-19 were recorded at the time of testing.

### Testing Procedures

All testing was performed according to the manufacturers' IFU for Veritor (targets the nucleoprotein antigen of SARS-CoV-2; indicated for use within 5 days from symptom onset) and the reflex RT-PCR assays, and per the clinical laboratory standard operating procedure. Following Veritor testing during screening, indications for reflex to RT-PCR were as follows: (1) a positive RAT result, associated with either close contact with a positive individual or with some symptoms, was considered positive and not reflexed to RT-PCR; (2) a positive RAT test associated with asymptomatic individuals without close contact with a positive individual was reflexed to a RT-PCR test; (3) a negative RAT result associated with symptoms, or close contact with a positive individual, was reflexed to RT-PCR; and (4) a negative RAT result from an asymptomatic individual, without close contact with a positive individual, was considered negative and not reflexed to RT-PCR. Antigen tests were performed on-site, whereas RT-PCR testing (specific assay varied by laboratory per SOC) was conducted by commercial laboratories, as described previously by Rane et al. (20). Overall, a total of 384,118 individuals had available reflex RT-PCR results and the percentage of antigen test results that agreed with RT-PCR results was determined. Informed consent was provided by all study participants. Data collection associated with this study was approved by the Institutional Review Board of the City University of New York Graduate School of Public Health and Health Policy.

### Data Analysis

The diagnostic performance of the antigen test, including sensitivity, specificity, positive predictive value (PPV), and negative predictive value (NPV), was first evaluated using the available paired RT-PCR results. Estimation of the true diagnostic performance of the antigen test was further conducted based on the projection of the RT-PCR result to all subjects without paired RT-PCR results available. The projection was modeled by implementing two separate assumptions: Assumption 1 (worst antigen diagnostic performance estimates): Individuals with confirmatory RT-PCR results in a certain symptom category (i.e., symptomatic vs. asymptomatic) are similar to (representative and reflective of) the individuals with missing RT-PCR results and, thus, were projected as similar results in the individuals without RT-PCR results. Assumption 2 (best antigen diagnostic performance estimates): individuals without confirmatory RT-PCR results in a certain symptom category had a clinical picture in agreement with the antigen result, and RT-PCR (if performed) would have agreed with the antigen test.

Verification bias adjustment (VBA) involved data imputation and was utilized to normalize for the difference in the rate of selection for RT-PCR testing between individuals, with positive and negative RAT results (21). The R statistical software package version 4.1 was used to perform data analysis for this study.

Performance values (sensitivity, specificity, PPV, and NPV) were determined using standard statistical methods. Post-test probability of being SARS-CoV-2-positive after a positive RAT was calculated as follows:

Pre-test probability (prevalence) was converted to pre-test odds (=[pre-test probability]/[1-pre-test probability]); pre-test odds were then multiplied by the positive likelihood ratio (=[sensitivity]/[1-specificity]) to generate post-test odds; finally, post-test odds were converted to post-test probability (=[post-test odds]/[1+post-test odds]).

Post-test probability of being SARS-CoV-2-positive after a negative RAT was calculated as follows:

Pre-test probability (prevalence) was converted to pre-test odds (=[pre-test probability]/[1–pre-test probability]); pre-test odds were then multiplied by the negative likelihood ratio (=[1–sensitivity]/[specificity]) to generate post-test odds; finally, post-test odds were converted to post-test probability (=[post-test odds]/[1+post-test odds]).

This article was prepared according to STARD guidelines for diagnostic accuracy studies reporting (22).

## Results

### Antigen Test Positivity and Reflex RT-PCR Testing

The demographic makeup of this study population was described previously by Rane et al. (20). Of the 1,952,931 antigen tests included in this analysis, 143,388 (7.3%) were positive, regardless of symptom presentation (Table 1). A total of 384,118 individuals (20%) had an available RT-PCR result (positive or negative). Of the available, paired RT-PCR results for symptomatic individuals, 94.4% agreed with the positive RAT results and 90.6% agreed with negative RAT results. Of the available paired RT-PCR results for asymptomatic individuals, 83.4% agreed with the positive RAT results and 95.3% agreed with negative RAT results. Overall, 93.1% of RAT results agreed with the paired RT-PCR results, regardless of symptom status.

**Table 1:** Percent agreement for specimens with a reflex RT-PCR test after antigen test for SARS-CoV-2.

**Symptomatic**	**Antigen**	***N* (% subtotal or total)**	**PCR Available (% of *N*)**	**PCR POS**	**PCR NEG**	**% of Veritor RAT results confirmed**
Yes	POS	86,811 (25.3%)	4,518 (5%)	4,265	253	94.4%
	NEG	256,442 (74.7%)	154,318 (60%)	14,559	139,759	90.6%
	Subtotal	343,253	158,836	18,824	140,012	
No	POS	53,046 (3.4%)	8,024 (15%)	6,693	1,331	83.4%
	NEG	1,506,687 (96.6%)	207,684 (14%)	9,729	197,955	95.3%
	Subtotal	1,559,733	215,708	16,422	199,286	
Missing	POS	3,531 (7.1%)	266 (8%)	222	44	83.5%
	NEG	46,414 (92.9%)	9,308 (20%)	541	8,767	94.2%
	Subtotal	49,945	9,574	763	8,811	
Total population	POS	143,388 (7.3%)	12,808 (9%)	11,180	1,628	87.3%
	NEG	1,809,543 (92.7%)	371,310 (21%)	24,829	346,481	93.3%
	Total	1,952,931	384,118	36,009	348,109	93.1%

*RT-PCR, reverse transcriptase-polymerase chain reaction; POS, positive; NEG, negative; RAT, rapid antigen test*.

### Estimated Diagnostic Performance

**Figure 1:**
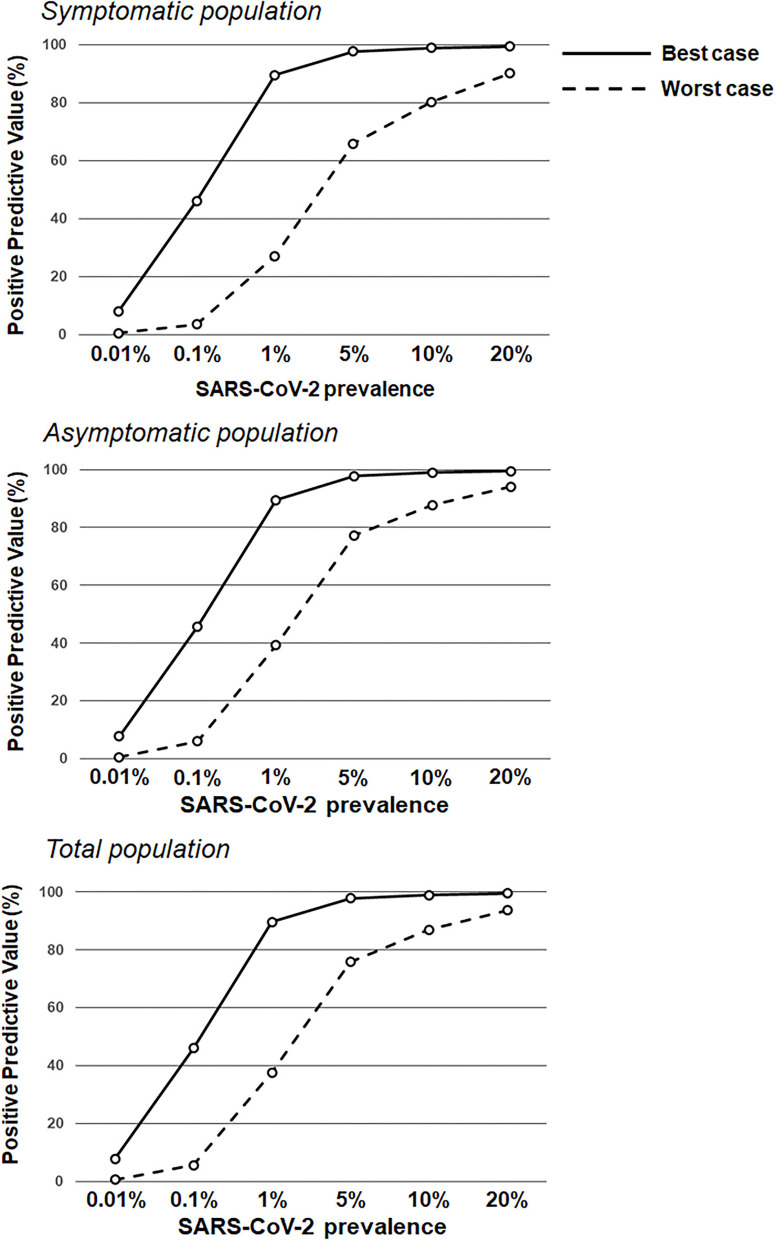
Positive predictive values based on RAT sensitivity/specificity over a range of theoretical SARS-CoV-2 prevalence values. RAT positive predictive value estimates in the symptomatic population (**top**; sensitivity of 77.2 and 85.6% in the worst antigen diagnostic performance estimates and best antigen diagnostic performance estimates, respectively; and specificity of 97.9 and 99.9% in the worst antigen diagnostic performance estimates and best antigen diagnostic performance estimates, respectively) and the asymptomatic population (**middle**; sensitivity of 38.5 and 84.2% in the worst antigen diagnostic performance estimates and best antigen diagnostic performance estimates, respectively; and specificity of 99.4 and 99.9% in the worst antigen diagnostic performance estimates and best antigen diagnostic performance estimates, respectively), and the total population (**bottom**; sensitivity of 53.8 and 85.1% in the worst antigen diagnostic performance estimates and best antigen diagnostic performance estimates, respectively; and specificity of 99.1 and 99.9% in the worst antigen diagnostic performance estimates and best antigen diagnostic performance estimates, respectively) based on increasing prevalence values (0.01–20.0%).

**Figure 2:**
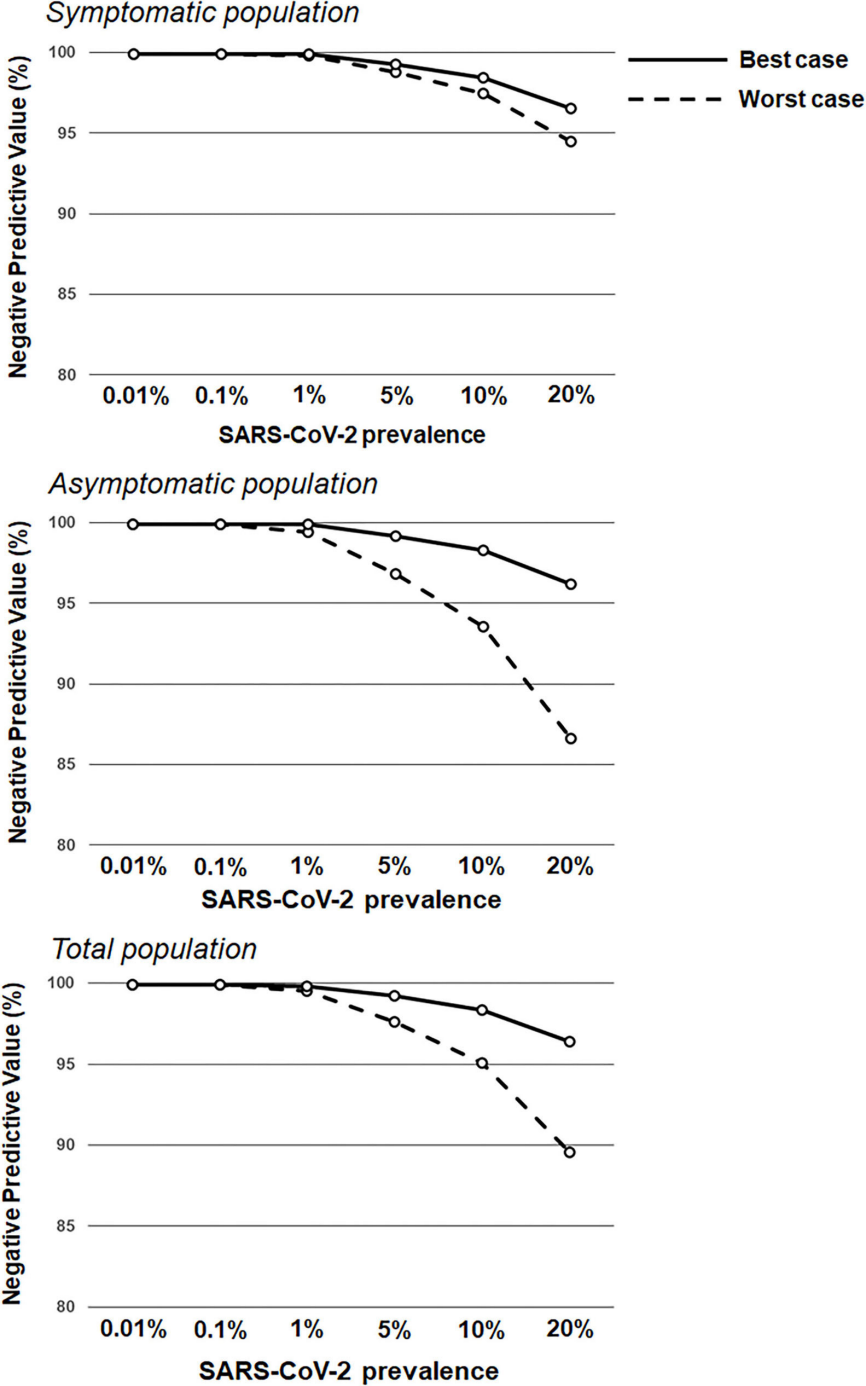
Negative predictive values based on RAT sensitivity/specificity over a range of theoretical SARS-CoV-2 prevalence values. RAT negative predictive value estimates in the symptomatic population (**top**; sensitivity of 77.2 and 85.6% in the worst antigen diagnostic performance estimates and best antigen diagnostic performance estimates, respectively; and specificity of 97.9 and 99.9% in the worst antigen diagnostic performance estimates and best antigen diagnostic performance estimates, respectively) and the asymptomatic population (**middle**; sensitivity of 38.5 and 84.2% in the worst antigen diagnostic performance estimates and best antigen diagnostic performance estimates, respectively; and specificity of 99.4 and 99.9% in the worst antigen diagnostic performance estimates and best antigen diagnostic performance estimates, respectively), and the total population (**bottom**; sensitivity of 53.8 and 85.1% in the worst antigen diagnostic performance estimates and best antigen diagnostic performance estimates, respectively; and specificity of 99.1 and 99.9% in the worst antigen diagnostic performance estimates and best antigen diagnostic performance estimates, respectively) based on increasing prevalence values (0.01–20.0%).

Based on underlying assumptions, two performance scenarios (see Methods and Materials) were generated for participant subgroups (symptomatic and asymptomatic). For the symptomatic population, the worst antigen diagnostic sensitivity and specificity estimates were 77.2 and 97.9%, respectively, and corresponded to 94.4% PPV and 90.6% NPV; the best antigen diagnostic sensitivity and specificity estimates were 85.6 and 99.9%, respectively, which corresponded to 99.7% PPV and 94.3% NPV (Table 2; Figures 1, 2). For the asymptomatic population, the worst antigen diagnostic sensitivity and specificity (Assumption 1) estimates were 35.8 and 99.4%, respectively, which corresponded to 83.4% PPV and 95.3% NPV; the best antigen diagnostic sensitivity and specificity (Assumption 2) estimates were 84.2 and 99.9%, respectively, and corresponded to 97.5% PPV and 99.4% NPV.

**Table 2:** Antigen test diagnostic performance under different assumptions for the specimens without paired RT-PCR results.

**Description**	**Antigen prevalence (%)**	**RT-PCR prevalence (%)**	**Sensitivity (%)**	**Specificity (%)**	**PPV (%)**	**NPV (%)**
**Symptomatic (*****n*** **=** **343,253)**						
Worst case analysis	25.3	30.9	77.2	97.9	94.4	90.6
Best case analysis		29.5	85.6	99.9	99.7	94.3
**Asymptomatic (*****n*** **=** **1,559,733)**						
Worst case analysis	3.4	7.4	38.5	99.4	83.4	95.3
Best case analysis		3.9	84.2	99.9	97.5	99.4
**Total population (*****n*** **=** **1,902,986)**						
Worst case analysis	7.3	12.1	53.8	99.1	88.7	94.0
Best case analysis		8.5	85.1	99.9	98.9	98.6

*RT-PCR, reverse transcriptase-polymerase chain reaction; PPV, positive predictive value; NPV, negative predictive value*.

**Figure 3:**
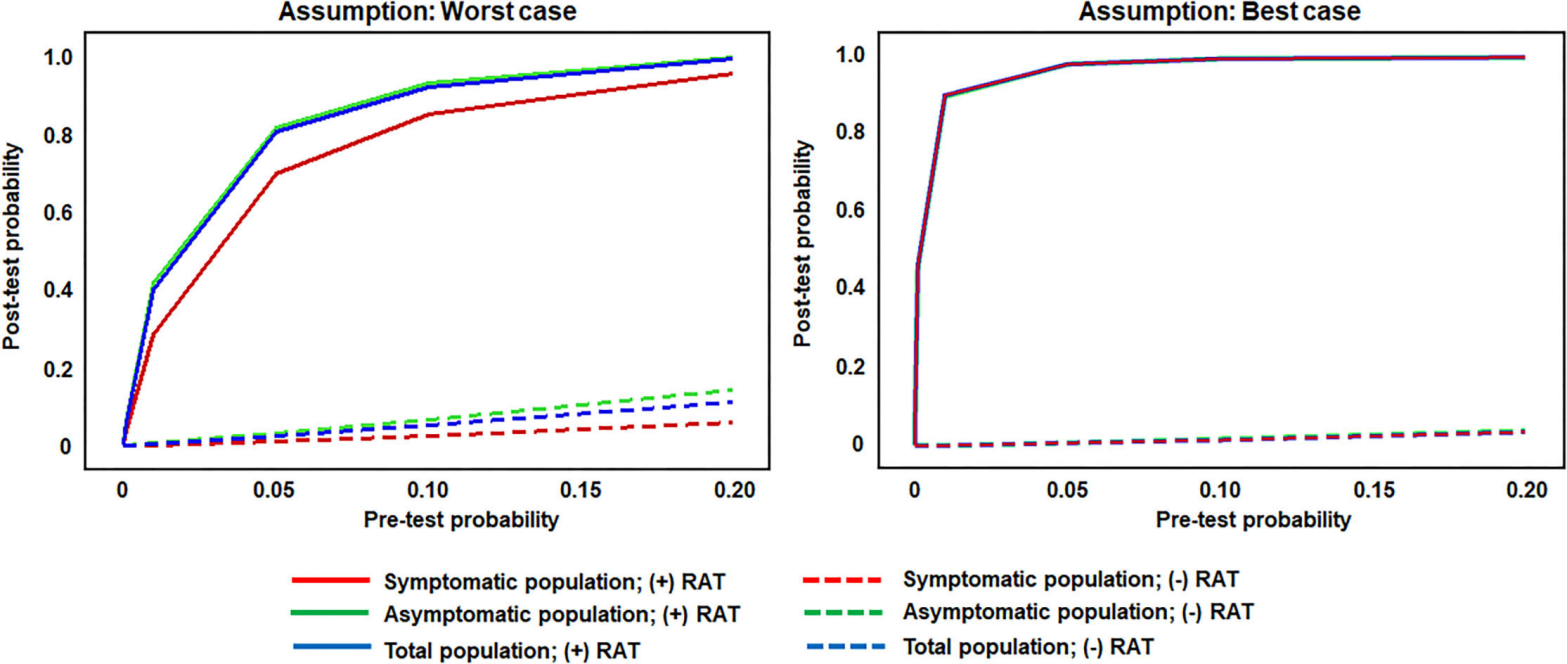
Post-test probability values for RAT-positive and -negative results across theoretical pre-test probabilities (prevalence values) and utilitzing set sensitivity and specificity values. RAT pre-test and post-test probability of infection in the symptomatic population (sensitivity of 77.2 and 85.6% in the worst antigen diagnostic performance estimates and best antigen diagnostic performance estimates, respectively; and specificity of 97.9 and 99.9% in the worst antigen diagnostic performance estimates and best antigen diagnostic performance estimates, respectively), the asymptomatic population (sensitivity of 38.5 and 84.2% in the worst antigen diagnostic performance estimates and best antigen diagnostic performance estimates, respectively; and specificity of 99.4 and 99.9% in the worst antigen diagnostic performance estimates and best antigen diagnostic performance estimates, respectively), and the total population (sensitivity of 53.8 and 85.1% in the worst antigen diagnostic performance estimates and best antigen diagnostic performance estimates, respectively; and specificity of 99.1 and 99.9% in the worst antigen diagnostic performance estimates and best antigen diagnostic performance estimates, respectively). RAT, rapid antigen test.

Post-test probabilities for a positive SARS-CoV-2 status following either a positive or negative RAT, for both model assumptions (worst and best performance estimates), are shown in Figure 3. Two major inflection points were observed in the worst antigen performance estimate for individuals with a positive RAT. A rapid increase in post-test probability was seen for all groups between pre-test probability (prevalence) values of 0–0.2; this was followed by a reduced rate of increase between 0.2 and 0.6; the slope then flattened from 0.6 to 0.10 and stayed relatively flat. Post-test probability values for individuals with a negative RAT remained below 0.2 across all pre-test probability values. For the best antigen performance estimate, post-test probability values associated with RAT-positive individuals rose sharply between pre-test probability values of 0–0.2, for a post-test probability of 0.90, while post-test probability values for RAT-negative individuals stayed below 0.10 across all pre-test probability values. For both assumptions, across a pre-test probability range of 0.075–0.10, the post-test probability for a positive SARS-CoV-2 status in both symptomatic and asymptomatic populations was ≥0.80 following a positive RAT, whereas the post-test probability for a positive SARS-CoV-2 status was below 0.10 following a negative RAT.

## Discussion

This real-world evidence study determined the performance of the Veritor RAT, during SARS-CoV-2 screening for over 1.95 million individuals, across multiple CityMD urgent care centers in the New York metropolitan area. Although reflex RT-PCR testing was not performed for every RAT result, the reflex RT-PCR test results that were obtained showed a high percent (93.1%) agreement with RAT results from the total population. Overall, the results here are consistent with the general performance characteristics of SARS-CoV-2 RATs that have been demonstrated previously (16, 18). Depending on the assumption for reflex RT-PCR testing that was applied, the sensitivity value for the RAT was either 77.2% (Assumption 1) or 85.6% (Assumption 2) in the symptomatic population, which overlaps with the sensitivity estimate [84% (95%CI: 67%, 93%)] listed for symptomatic individuals in Veritor's instructions for use (23). Consistent with the performance of other RATs, Veritor testing here was associated with a relatively low false-positive rate and high specificity. For low SARS-CoV-2 prevalence (0.001–5%), the results here show a range of RAT PPVs, depending on the applied assumption for reflex RT-PCR testing and depending on the absence or presence of symptoms. However, as prevalence increased to 10%, PPVs for the best- and worst-case reflex RT-PCR assumptions rise to ≥80% (Figures 1, 3) in both the symptomatic and asymptomatic populations, which is consistent with previous work (13, 24–26). RAT NPV here was >90% when SARS-CoV-2 prevalence was between 7.5 and 10%, and did not drop below 90% until prevalence exceeded 20%, providing further confirmation that a negative RAT result provides good assurance of a negative status at a 10% SARS-CoV-2 prevalence (Figure 2).

Rapid antigen testing has a lower analytical sensitivity compared to nucleic acid amplification testing, which translates to a lower clinical sensitivity when compared to molecular testing-based reference assays (13). RAT sensitivity is significantly better when performed on upper respiratory specimens with a viral load between 1X10_5 and 1X10_6 genomic copies/mL (corresponding to a Ct score of ~ ≤30 and ≤25, respectively, depending on the reference RT-PCR assay employed) (18, 27–32). Therefore, these findings regarding the variability in RAT sensitivity, when stratified by the presence or absence of COVID-19 symptoms, are not surprising given that symptomatic individuals are typically associated with specimens that have a higher viral load. The results here are consistent with previous studies that have shown better sensitivity of SARS-CoV-2 RATs when specimens are obtained from symptomatic individuals (16, 18). However, although RAT sensitivity is somewhat reduced when compared to molecular-based testing, several drawbacks are associated with molecular testing including limitations in specimen processing capacity (33, 34), prolonged turnaround time (at best 24 hours when sample shipment is considered), and the need for dedicated staff and automated platforms; all of which can limit turnaround time and impede optimized patient management (35). Reagent and collection swab shortages can also limit the capacity associated with molecular-based testing (36, 37).

RATs represent an efficient and cost-effective means for SARS-CoV-2 testing in both symptomatic and asymptomatic screening populations. Love et al. recently demonstrated that frequency of testing, regardless of whether RT-PCR or rapid antigen testing is employed, in combination with effective quarantine, can provide an effective strategy to suppress the spread of SARS-CoV-2 (12). In real-world settings, the need for quarantine could be identified sooner following performance of a RAT, compared to a RT-PCR test, with a reflex RT-PCR test used to confirm the RAT result. In addition, serial testing with RATs should be easier to implement successfully due to their faster turnaround time. Ultimately, multiple factors, including the duration of symptoms, previous screening results, and the screening setting, need to be weighed to determine whether a RAT should be employed to preclude molecular-based testing (38). Regardless, further evidence is needed to confirm RAT sensitivity estimates that are listed in manufacturer IFUs to ensure that the RAT in question performs as indicated in a real-world setting (19). Allan-Blitz et al. recently demonstrated a notably lower sensitivity estimate for one RAT in a large study population than that originally listed in the manufacturer's IFU (15). In addition, several studies, including some meta-analyses, have shown variability in RAT sensitivity, depending on several study-related factors. Trials and studies performed for product registration can involve several forms of bias that improve the likelihood of an expected outcome (39). These include reporting bias (e.g., reporting RAT performance in a manner that is stratified by lower cycle threshold score [higher viral load]) or selection (spectrum) bias (e.g., proportion of symptomatic and asymptomatic individuals), both of which can artificially increase the number of specimens with a high viral load in the final analysis (40–42).

Since the end of 2020, several SARS-CoV-2 variants have been designated by the World Health Organization that display certain characteristics, including increased transmissibility, increased virulence, and/or association with decreased effectiveness of public health measures (43). Globally, increased COVID-19 cases have been observed, due mainly to the increased prevalence of SARS-CoV-2 variants, especially the Delta variant (44–46)—even in countries such as the US (4) and the UK (47, 48), which have relatively high COVID-19 vaccination rates. This situation emphasizes the importance of accessibility to rapid and reliable SARS-CoV-2 testing, as a means to identify positive individuals quickly and mitigate community transmission. The manufacturer of Veritor has established a robust testing program to continuously evaluate the performance of this RAT assay on new and evolving SARS-CoV-2 variants of concern. As a preliminary means of testing, the manufacturer has utilized *in-silico* analysis to identify key mutations within the nucleocapsid coding region. Once these mutations are identified, additional testing of live, or heat inactivated isolates, is performed to confirm detection. As of December 2021, the manufacturer of Veritor has not identified any SARS-CoV-2 variants with nucleocapsid mutations that impose significant detection or assay performance deficits. Utilizing the testing program described above, the manufacturer of Veritor has so far confirmed detection of the Alpha, Beta, Gamma, Kappa, Iota, Delta, Lambda, Mu, and Omicron variants (Supplementary Table S1). However, it will be important to perform new studies for Veritor and other RATs, either as part of registration submissions to regulatory agencies, or as part of real-world investigations, to determine accurate performance estimates for SARS-CoV-2 variants as they are discovered.

### Limitations

This real-world study is associated with certain limitations encountered during design, conduct, and analysis. The exact reflex RT-PCR test paired with each Veritor test result was not captured for this data set. Therefore, it is possible that subsets of the data set were matched with RT-PCR tests that have different analytical sensitivities. At relatively low viral loads, this could have affected “clinical truth,” and differentially led to RAT false-negative results in certain cases but true negative results in others. However, a meta-analysis that included over 80 RAT studies previously showed that the analytical sensitivity of the reference RT-PCR tests does not affect RAT performance in a statistically significant manner (18). In addition, the specimen collection procedure (e.g., nasal vs. nasopharyngeal swab) or the storage conditions (e.g., fresh vs. frozen) for reflex RT-PCR testing could have differed for individual RT-PCR tests in this data set, which could have impacted overall RAT agreement. Also, the modeling applied here for missing reflex RT-PCR tests may not have accurately reflected the distribution of actual reflex RT-PCR results that would have been obtained. For this particular study, missing-at-random was considered the worstcase for the missing data sensitivity analysis as described in Campbell et al. (49). While it is reasonable to assume that clinicians were just as likely to reflex individuals with lower and higher RT-PCR agreement (based on clinical picture); mathematically it is not possible to prove *worst case*. Finally, although there is no evidence that Veritor was used in a manner inconsistent with the manufacturer's IFU by CityMD clinics in this study, it is possible that some specimen collection procedures could have deviated from those outlined in the Veritor IFU.

## Conclusions

This was a real-world analysis involving rapid antigen testing that included ~1.95 million test results from a network of urgent care clinics in the New York metropolitan area. While these results are dependent on assumptions and data imputation in order to assess aspects of RAT performance, in general they show that Veritor sensitivity is consistent with that listed in the manufacturer IFU. The specificity for the RAT was >95%, regardless of the testing population or assumptions that were applied during data analysis. In addition, these data show that the RAT has effective positive and negative predictive values in a real-world screening population, for both symptomatic and asymptomatic individuals. The performance of RATs should continue to be evaluated in real-world situations to rigorously assess their value during screening, especially as SARS-CoV-2 variants continue to develop and spread.

## Data Availability Statement

The original contributions presented in the study are included in the article/Supplementary Material, further inquiries can be directed to the corresponding author/s.

## Ethics Statement

The studies involving human participants were reviewed and approved by the Institutional Review Board of the City University of New York Graduate School of Public Health and Health Policy. Written informed consent from the participants' legal guardian/next of kin was not required to participate in this study in accordance with the national legislation and the institutional requirements.

## Author Contributions

VP, DF, KL, and CC: experimental design and data interpretation. LC: data interpretation. AP: experimental design, experimental implementation, and data interpretation. All authors contributed to the interpretation of the data, critically revised the manuscript for important intellectual content, approved the final version to be published, and agree to be accountable for all aspects of the work.

## Funding

This study was supported, in part, by Becton, Dickinson and Company; BD Life Sciences—Integrated Diagnostic Solutions.

## Conflict of Interest

VP and LC are employees of Becton, Dickinson and Company. CC was an employee of Becton, Dickinson and Company at the time of preparation for this manuscript. AP, DF, and KL are employees of Summit Health, which is the parent company of CityMD.

## Publisher's Note

All claims expressed in this article are solely those of the authors and do not necessarily represent those of their affiliated organizations, or those of the publisher, the editors and the reviewers. Any product that may be evaluated in this article, or claim that may be made by its manufacturer, is not guaranteed or endorsed by the publisher.
