# Supplementary material for: Large-Scale SARS-CoV-2 Antigen Testing With Real-World Specimens
Source: Front Public Health. 2022 Apr 5;10:836328. doi: 10.3389/fpubh.2022.836328 (PMC9016156; doi:10.3389/fpubh.2022.836328)
Supplement: Supplementary file 1 [file Data_Sheet_1.PDF]

Table S1. Live or heat-inactivated SARS-CoV-2 variant isolates with confirmed detection by the Veritor antigen test

WHO Nomenclature	Variant	Reference Isolate Tested
Alpha	B.1.1.7	SARS-Cov-2, Isolate USA/CA_CDC_5574/2020, BEI Resources Catalog No. NR-54011
Beta	B.1.351	SARS-Cov-2, Isolate hCoV-19/South Africa/KRISP-K005325/2020, BEI Resources Catalog No. NR-54009
Gamma	P.1	SARS-Cov-2, Isolate hCoV-19/Japan/TY7-503/2021, BEI Resources Catalog No. NR-54982
Kappa	B.1.617.1	SARS-Cov-2 Isolate hCoV-19/USA/CA-Stanford-15_S02/2021, BEI Resources Catalog No. NR-55486
Iota	B.1.526	SARS-Cov-2 Isolate hCoV-19/USA/NY-NP-DOH1/2021, BEI Resources Catalog No. NR-55359
Delta	B.1.617.2	SARS-Cov-2 Isolate hCoV-19/USA/PHC658/2021, BEI Resources Catalog No. NR-55611
Lambda	C.37	U.S. NIH RADx Tech Team 6496 Screening Panel (Sept 23, 2021)
Mu*	B.1.621	U.S. NIH RADx Tech Team 6496 Screening Panel (Sept 23, 2021)

* The Mu variant (B.1.621), contains the same characteristic T205I mutation as the Beta variant
